# Politics and Psychology

**DOI:** 10.1007/s12124-023-09802-y

**Published:** 2023-09-11

**Authors:** Peter Busch-Jensen, Maja Røn-Larsen

**Affiliations:** Department of People and Technology, Roskilde University, Roskilde, Denmark

**Keywords:** Psychology of everyday life, Political psychology, Everyday politics, Critical social psychology, Cultural psychology

## Abstract

This article presents a discussion inspired by the invitation formed by Kevin Carriere’s book: “Psychology in Policy – Redefining Politics Through The Individual”. From a theoretical standpoint in culture psychology Carriere challenges the idea of politics as a particular practice carried out by mainly politicians. Instead, he attempts to anchor processes of politics in the everyday lives of individuals, directed at changing their worlds. In this article, we discuss how this ambition could evolve even further by relating it to other theoretical approaches working with similar ambitions.

## Introduction

There is a new book out. “Psychology in Policy – Redefining Politics Through The Individual”, by psychologist Kevin Carriere. The book is short, less than 100 pages, with a preface by Giuseppina Marsico and Jaan Valsiner. In both content and form, it tends to represent more of a manifest, than a research report. As such, Carriere’s book is an invitation to the community of psychological researchers to revisit the central topic about politics of everyday lives of people – opening up discussions on how to understand human activities as immanently political, related to participation in transforming our common world, which again leads to deeper reflections on the dialectical person-structure relations. In this short article, we accept the invitation and will attempt to discuss future possibilities for developing the discussion further by drawing on cognate perspectives from other disciplines and theoretical bases.

As implied by the title, Carriere sets out to challenge common understandings of politics as simply a matter for politicians. Hence, inspired by the work of Valsiner and culture psychology, he develops an alternative approach, namely politics understood as a result of human social interaction in more general cross-contextual and inter-disciplinary terms. Obviously, it is important to study politics as a specific domain, craft and social practice. However, it is equally important to understand politics in broader terms, not as a particular craft carried out by specific professionals and actors in particular institutional settings and arenas, but as something resulting from a of ‘myriad of actions’ done by ‘many different people in ways that are happening more or less unnoticed’ (Carriere, 2022: xiii). It is this latter understanding Carriere sets out to argue for, describe and explore. Along the way, he furthermore invites his reader to notice some broader implications for how we understand human psychology and social institutions.

## Psychology in Policy – Redefining Politics Through the Individual

The main goal for Kevin Carriere is to anchor political processes in people’s everyday lives and personal processes of meaning making. Thus, an implicit ambition is to address the persons involved in science as more than just objects of research; and to include them instead as active meaning-creating agents, that researchers need to study *with* rather than *on*. Building on a framework in cultural psychology Carriere attempts to link cultural psychology, public policy and political psychology, in order to overcome, what he identifies as a theoretical gap in our understanding of the cultural meaning making processes behind the formation of political beliefs, institutions and activities. Elaborating on this ambition, Carriere defines political psychology as “the psychological study of the intersections of competing values, policies, and power dynamics” (Carriere, 2022: 5) and suggest we center the analysis on “the individual and their own meaning-making processes” (ibid.). As Carriere state his focus: “There is an agentic quality to holding political beliefs. (…) Our values do not simply provide us with direction of what political party to back, or who we elect to a local township office, but can direct our individual activism behavior. We take action to make changes for ourselves (…) we work to make changes for our own future” (Ibid: 6).

Carriere’s argumentation is inspired by a 1 year-long empirical study based on a post-doctoral fellowship, where Carriere had the opportunity to work for Congresswoman Debra Haaland of New Mexico’s first district.

The latter is used to study the large unrecognized pre-legislative work made by the staff “behind” the politician. As it turns out, this work is tied up in culture, values, norms and meaning-making processes of people’s everyday life, which in numerous unnoticed ways effects the political processes and results. Carriere uses this observation to engage the reader in a larger discussion on the difference between our official imaginary of politics as based in science and run by publicly elected politicians, and a hidden reality of politics and policy influenced by culture, norms, values, interests and individual non-elected members of for example staff-members and others.

His analysis starts with the fact that politicians to a large extent employ staff members to do the legislative work. The interesting thing here is how staff members of course have their own special cases and political agendas that are rooted in personal cultural and historical contexts, but that these are never made transparent or explored as such. Carriere draw a parallel to psychology, since just as the pre-legislative work behind the policy reveal a surprising influence and power from non-elected staff-members, one will also find surprising influences from processes of culture, value, norms and meaning-creation behind so-called scientific findings within psychology. Carriere offers The Stanford Prison experiment, by psychologist Philip Zimbardo, as an illustrative example of how the values of the researcher play into the research process and its findings.

The argument amounts to a more general critique of mainstream notions of not only politics but also of science. It is a well-known critique that science is a human activity, which is always - in one way or the other - entangled in values, culture, and special interest. This critique, however, is often addressed in ways that ignore the wider issue, by discarding the interplay between ideal and reality as simply a result of bad science; in that way turning the critique into a call for more scientific rigor and objective dealings with knowledge. By pointing to the inevitability of such entanglements as a starting point for a psychology of politics, Carriere describe the problem as more fundamental and argues for a psychology, that takes point of departure in the process instead of the product, and the involved individuals instead of the institution.

Carriere builds up his argument by pursuing a selected set of topics: Values; The intersection of the private and the public, and finally power and policy, - each discussed in more detail in three separate chapters. Together the chapters add up to a description of political life from a more mundane, everyday life perspective of not only politicians, but also legislators and others that work and influence politics from ‘behind the scene’. This is both needed and important, compared to much mainstream psychology and political discourse. Furthermore, it is done in ways that invite the reader to consider and reimagine what not only politics but also psychology could turn out to be, if we focused on the stories instead of statistics, the process instead of the product, and the individual instead of the institution. Thus, an important quality of the book, is how it invite readers to take the individual - the patient, the voter, the citizen -, more serious, as not simply an object of politics or psychology, but an active agent of both.

## Discussion

Carriere emphasize human agency as a dynamic individual orientation towards value and meaning. Doing so, he implicitly leans into several larger traditions within social science and humanities, such as hermeneutics, phenomenology, post-structuralism, critical theory and activity theory; but for some reason this is not made very explicit, nor thoroughly reflected upon in the argumentation. As readers, this left us with a number of unanswered questions and sense of missed opportunities for interesting discussions.

In the following, we will address some of these issues. Firstly, we will point to the possibilities of continuing a fruitful dialogue, with like-minded work within other disciplines as well as perspectives already developed within psychology. On this background, we will pose some questions to Carriere’s book as such, discussing whether the presented theoretical perspective is in fact followed through in the books form, method, and argumentation. The aim is to contribute to the further development of the theoretical discussions on the relation between politics and psychology.

According to Carriere, the unique quality of the book is its “innovative theoretical analysis of how political systems are being set to function between societal structures that set up the frame of “politics” and individuals who establish their particular social role relations with that frame” (Carriere, 2022: V). What matters is therefore “understanding the process that occurs when the individual is engaging with policy in their daily life” (Ibid: xiii). Again, this is a timely and relevant focus in today’s landscape of crisis within many democracies. However, it is far from being as unique as is claimed by Carriere and the editors of the book.

In the editors preface, it is claimed, that the question on how politics are “the result of human inventions – such as technology or religions” has not been covered in psychology, until “cultural psychology came to the scene” (Marsico & Valsiner, 2022: v). Appreciating the contribution from cultural psychology, such a statement seems to underestimate existing discussions within psychology as well as related disciplines. We will begin with the latter.

The book present “a psychology that studies the intersection of values, power dynamics, and public policy” (Carriere, 2022: xiv), from an observation that this is an overlocked relationship within psychology. This might be true, if we look at standard notions of psychology in public discourse. However, it is not true that psychology as such, nor the social sciences in general for that matter, have neglected human agency and its role in the intersection between politics and psychology. There is a rich line of inquiry in social psychology and other disciplines, which represents different attempts to unpack the complex processes by which individuals actively participate in the co-construction of social life and its structural dimensions such as ideological beliefs, politics, and policy. In fact, there is such a substantial body of theorizing on this and on why and how we need to include the standpoint of the subject in our understandings of these dimensions, that this field is rather marked by balkanization, than neglect (Ibáñez & Íñiguez, 1997; Holzkamp, 2013, 2016; Stetsenko, 2008, 2013).

Carriere’s interest in lines of thought outside culture psychology is surprisingly absent, and this weakens his argumentation. For example, as we suspect will also surprise other readers, the book pursues an ambition to explore the connection between politics and psychology without a single mention of Michel Foucault’s work and the thick body of work it has inspired within psychology on precisely the connection between politics and subjectivity (Foucault, 1978, 1980, 1982). This is a conspicuous absence, since much of this work focus on the very processes of value-, culture-, and meaning-creation, which Carriere examines (See e.g. Butler, 1997; Haraway, 1991; Rose, 1989, 1996, 2007; Flyvbjerg, 1998).

Like Carriere, the Foucauldian tradition centers on the entanglement of politics, science and subjectivity, but from a wider critical attention to how late-modern democracies are being cast inside particular divisions of labor between politics and science, which pertain also to particular knowledge-hierarchies and processes of subjectivity, norms and value. Obviously, it would lead too far to go into detail with the Foucauldian perspective here. The point is that it has contributed to critical self-reflection on the researcher’s own apparatus and its potential blind spots: an a healthy hesitation towards the clarity and solidity of the categories with which we conceptualize our object, the methodological approaches with which we try to meet and grasp the world and the organizational framework in which all this takes place.

Carriere finds himself part of a *particular* top-down form of governance in which policies are produced in a *certain* electoral system, by elected politicians that collaborate on pre-legislative work with a hired staff of professionals. However, might democracy not be something very different? Could it not be more direct, bottom-up or decentralized in the first place? Where does this particular organizational setup come from and what does it mean for how one can study politics and for how politics and psychology are entangled in one’s study? Carriere does not ask these questions. As a result, he explores the people, stories, processes and voices he encounters without asking what stories and who’s experiences might *not* be there, and maybe even conspicuously absent or silent. Could there be something absence in the present, which might be central for a political psychology to address? This seems a relevant question to ask, when one express an ambition to study “the intersections of competing values, policies, and power dynamics” (Carriere, 2022, p.5).

Another example relates to Carriere mentions of Aristoteles and his claim of humans as political animals (Carriere, 2022, p. 21). Carriere takes this description as his starting point for how he thinks about the role and dimensions of politics in social life. However, again, he does not pursue if this line of thinking have maybe been pursued and developed also by others, and if useful questions might turn up in this tradition.

Hannah Arendt’s work on politics as an “acting in concert” is an obvious example (e.g. Arendt, 1958, 2005), also explored within psychology (Mori, 2003). In line with Carriere’s own perspective, politics, according to Arendt, is a domain of human plurality and difference. Therefore, political power is not simply about formal representation or the formation of a social consensus. Politics, on a more foundational level, arises from social processes in which the common is *produced* in common.

Arendt’s work has given rise to countless discussions, some of which Carriere could have usefully included. For example: if politics is also about *if* and *how* the common is *produced* in common, must a psychology of politics then not also address how we deals with differences, conflicts and dilemmas? Furthermore, if politics is about the production of a common world, can a psychology of politics isolate the study of meaning, value and experience, from the shared *worldly* activities they are part of, and that involves a joint creation of a common world, done by people who argue with one another from a common engagement in *specific* problems?

In continuation of this, critical psychology describes politics as *a worlding practice*, the processes through which the reciprocated relation between human communities and their societal conditions is developed (Busch-Jensen & Schraube, 2019; Holzkamp, 2013, 2016; Dreier, 2008; Schraube, 2024). We find a similar standpoint developed within Culture Historical Activity Theory, where e.g. the psychologist Anna Stetsenko has proposed the Transformative Activist Stance (TAS) as an attempt to emphasize people as political activists always simultaneously situated in and directed at transforming the world to a better place (Stetsenko, 2008; Højholt & Røn-Larsen, 2021, Røn-Larsen, 2019, 2023). Stetsenko insists on the individual as always inherently societal and cultural, simply by being part of the world and at the same time transforming it through future-oriented agendas (Stetsenko, 2013).

Even though Carriere’s understanding of politics is not mainstream, neither is it all novel. The complex reciprocal relationship between the personal and the political is today a shared attention, both within much community psychology (Fisher et al., 2007; Hooks, 1994; Hope et al., 2018; Kloos et al., 2012; Rappaport, 1987; Macías-Gómez-Estern, 2021; Thai & Lien, 2019) and within much social psychology and feminist theory. Carriere could have noticed, for example, the famous claim within feministic traditions that “the private is political” – a claim brought into psychology by for example Frigga Haug (Haug, 1992, 2016) and elaborated on by numerous feminist theorists (Allan, 2008; Bachrach & Baratz, 1962; Nicholson et al., 1995; Brown, 1988; Crenshaw, 1991).

## Final Remark

Obviously, one cannot write a book about everything. That’s the name of the game. One must draw some boundaries. In Carrier’s book, however, we find some of these boundaries to have maybe been drawn a little too soon and too tight, sometimes weakening the books argumentation and potentially also the important cross-disciplinary theoretical development it invites its readers to engage in.

## References

[CR1] Allan A (2008). Power and the politics of difference: Oppression, empowerment, and transnational justice. Hypatia.

[CR2] Arendt H (1958). The Human Condition.

[CR3] Arendt, H. (2005). *The Promise of Politics*. Edited and with an introduction by Jerome Kohn. Schocken Books.

[CR4] Bachrach P, Baratz MS (1962). The two faces of power. American Political Science Review.

[CR5] Brown W (1988). Manhood and politics: A feminist reading in political theory.

[CR6] Busch-Jensen P, Schraube E, Højholt C, Schraube E (2019). Zooming in zooming out: Analytical strategies of situated generalization in psychological research. Subjectivity and knowledge: Generalization in the psychological study of everyday life.

[CR7] Butler J (1997). The psychic life of power: Theories in subjection.

[CR8] Carriere, K. (2022). *Psychology in policy - redefining politics through the individual.* SpringerBriefs.

[CR9] Crenshaw K (1991). Demarginalizing the intersection of race and sex: A black feminist critique of antidiscrimination doctrine. Feminist theory, and antiracist politics. University of Chicago Legal Forum.

[CR10] Dreier O (2008). Psychotherapy in everyday life.

[CR11] Fisher AT, Sonn CC, Evans SD (2007). The place and function of power in community psychology: Philosophical and practical issues. Journal of Community & Applied Social Psychology.

[CR12] Flyvbjerg B (1998). Rationality and power: Democracy in practice.

[CR13] Foucault M (1978). The history of sexuality.

[CR14] Foucault M (1980). Power/knowledge.

[CR15] Foucault M, Dreyfus H, Rabinow P (1982). The subject and power. Michel Foucault: Beyond structuralism and hermeneutics.

[CR16] Haraway, D. J. (1991). *Simians, cyborgs, and women: The reinvention of nature* (pp. 183–201). Routledge.

[CR17] Haug F (1992). Beyond female masochism: Memory-work and politics (questions for feminism).

[CR18] Haug F, Schraube E, Højholt C (2016). The politics of Hope: Memory-work as a method to study the conduct of everyday life. Psychology and the conduct of everyday life.

[CR19] Højholt C, Røn-Larsen M (2021). Conflicts, situated inequality and politics of everyday life. Culture & Psychology.

[CR20] Holzkamp K, Schraube E, Osterkamp U (2013). Psychology: Social self-understanding on the reasons for action in the conduct of everyday life. Psychology from the standpoint of the subject: Selected writings of Klaus Holzkamp.

[CR21] Holzkamp K, Schraube E, Højholt C (2016). Conduct of everyday life as a basic concept of critical psychology. Psychology and the conduct of everyday life.

[CR22] Hooks B (1994). Teaching to transgress.

[CR23] Hope EC, Velez G, Offidani-Bertrand C, Keels M, Durkee MI (2018). Political activism and mental health among black and latinx college students. Cultural Diversity and Ethnic Minority Psychology.

[CR24] Ibáñez T, Íñiguez L (1997). Critical social psychology.

[CR25] Kloos B, Hill J, Thomas E, Wandersman A, Elias M, Dalton J (2012). Community psychology: Linking individuals and communities.

[CR26] Macías-Gómez-Estern B, Suarez M (2021). Critical psychology for Community Emancipation: Insights from Socio-educative Praxis in Hybrid Settings. New waves in social psychology.

[CR27] Marsico, G., & Valsiner, J. (2022). Series Editors’ preface - politics brought home: cultural psychology of generating values. In: Carriere, K. (Ed.), *Psychology in Policy - Redefining Politics Through The Individual* (p. v - viii). SpringerBriefs.

[CR28] Mori T (2003). Plurality in “acting in concert”: A reexamination of Hannah Arendt’s concept of power. ARSP. Archives for Philosophy of Law and Social Philosophy.

[CR29] Nicholson, L. (Ed.). (1995). *Feminist contentions: A philosophical exchange*. Routledge.

[CR30] Rappaport J (1987). Terms of empowerment/exemplars of prevention: Toward a theory for community psychology. American Journal of Community Psychology.

[CR31] Røn-Larsen M (2019). Interdisciplinary collaboration and conflict concerning children in difficulties: Conditions, procedures and politics of everyday life in school. Annual Review of Critical Psychology (Online).

[CR32] Røn-Larsen, M. (2023). Everyday politics of educational psychologists. *Annual Review of Critical Psychology* (Online), 17.

[CR33] Rose N (1989). Governing the soul: The shaping of the private self.

[CR34] Rose, N. (1996). *Inventing our selves: Psychology, Power and Personhood*. Cambridge University Press.

[CR35] Rose N (2007). The Politics of Life itself: Biomedicine, Power, and subjectivity in the Twenty-First Century.

[CR36] Schraube E (2024). Why dialogue matters: Digitalization and learning as a worlding practice.

[CR37] Stetsenko A (2008). From relational ontology to transformative activist stance: Expanding Vygotsky’s (CHAT) project. Cultural Studies of Science Education.

[CR38] Stetsenko A (2013). The challenge of individuality in cultural-historical activity theory: “Collectividual” Dialectics from a transformative activist stance. OUTLINES.

[CR39] Thai, N. D., & Lien, A. (2019). Respect for diversity. In L. Jason, O. Glantsman, J. O’Brien & K. Ramian (Eds.), *Introduction to Community Psychology: Becoming an agent of change* (pp. 141 – 159). Creative Commons Atribbution.

